# Influenza Vaccination during Pregnancy: A Descriptive Study of the Knowledge, Beliefs, and Practices of Mexican Gynecologists and Family Physicians

**DOI:** 10.3390/vaccines11081383

**Published:** 2023-08-19

**Authors:** Erika Zoe Lopatynsky-Reyes, Enrique Chacon-Cruz, Michael Greenberg, Ralf Clemens, Sue Ann Costa Clemens

**Affiliations:** 1 Institute for Global Health, University of Siena, 53100 Siena, Italy; enrique.chacon@thinkvaccines.org (E.C.-C.); sue.costa@unisi.it (S.A.C.C.); 2 Think Vaccines LLC, Houston, TX 77005, USA; 3 Sanofi, Swiftwater, PA 18370, USA; michael.greenberg@sanofi.com; 4 International Vaccine Institute (IVI), Seoul 08826, Republic of Korea; clemens.ralf@outlook.com; 5 Department of Pediatrics, University of Oxford, Oxford OX1 2JD, UK

**Keywords:** influenza, influenza vaccination, pregnancy vaccination, physicians’ knowledge

## Abstract

Background: Influenza in pregnancy is associated with elevated morbidity and mortality. Influenza vaccines are safe and effective in pregnancy. There are no Mexican surveys of physicians on knowledge, beliefs, and practices towards influenza and influenza immunization during pregnancy. Methods: A 32-question descriptive survey was conducted, addressing the general knowledge of influenza as well as beliefs and practices regarding influenza vaccination during pregnancy among Mexican physicians responsible for prenatal care, traditionally Obstetricians (OBGYNs) and Family Physicians (FPs). Results: A total of 206 surveys were available, 98 (47.6%) from OBGYNs and 108 (52.4%) from FPs, representing an estimated 2472 daily pregnancy consultations. In total, 54 of the 206 respondents (26.2%) were not aware that influenza is more severe during pregnancy, 106 of the 206 respondents (51.5%) ignored the potential side effects of influenza infection on the fetus, and 56.8% did not know when to vaccinate pregnant women. Pregnancy as a risk factor for developing influenza complications was only known by 99 of the 206 respondents (48.1%), and 6.1% believed that vaccination does not confer protection to the fetus. Conclusions: The current beliefs of Mexican OBGYNs and FPs for both influenza morbidity and mortality, and the importance of influenza vaccination during pregnancy are suboptimal. The drivers of these beliefs should be assessed to improve influenza vaccination recommendations, as knowledge alone is not sufficient.

## 1. Introduction

Influenza is an acute viral respiratory disease that is highly contagious, transmitted through droplets, associated with seasonal antigenic drifts, and shifts with the risk of global pandemics. Influenza symptoms vary uniquely from person to person and can range from mild to severe in their presentation. The typical symptoms of influenza include fever, fatigue, cough, and body aches [1,2]. 

The virus reaches its peak contagion within the initial 3 to 4 days after symptoms appear, yet even before experiencing their own symptoms, healthy individuals can transmit the virus to others. Transmissibility can persist for up to 5 to 7 days, while symptoms may endure for up to 2 weeks or more [1,2]. The main preventive measure against influenza is vaccination, which is advised for everyone aged 6 months or older, including pregnant and postpartum women, unless there is a contraindication. Vaccination should occur at the start of the flu season, usually in October in the Northern Hemisphere. It takes approximately 14 days after vaccination for a healthy adult to achieve maximum antibody protection [2].

The main factors associated with higher morbidity and mortality from influenza are age (<5 years old or >65 years old), pregnancy, immunodeficiency, medical comorbidities, and genetic susceptibility [1,2]. 

Expectant mothers face a heightened susceptibility to influenza-related complications and are recognized as a prioritized demographic for both seasonal and pandemic flu immunization. After recent seasonal influenza outbreaks, it has become evident that pregnant women experience a significantly greater likelihood of hospitalization when compared to their non-pregnant counterparts [3]. During the third trimester of pregnancy, the rates of hospitalization peak, and expectant mothers are found to be 3–4 times more prone to hospitalization due to cardiopulmonary illnesses during the influenza season, in contrast to postpartum women [4,5]. 

Transplacental transmission of the 2009 AH1N1 virus was alleged in some cases, but definitive evidence was not available at the time. A recent study by Xiao et al. concluded that the human placenta can be infected [6,7]. Nonetheless, data from the AH1N1 pandemic highlight that influenza during pregnancy escalates the likelihood of unfavorable pregnancy outcomes. A study conducted on 256 women hospitalized in the United Kingdom in 2009 due to AH1N1 virus infection during pregnancy revealed a notable increase in the perinatal mortality rate (39 per 1000 total births (95% CI: 19–71)) among women with prenatal influenza, in comparison to 7 per 1000 (95% CI: 3–13) among the control group without 2009 H1N1 infection. Several risk factors for preterm delivery were identified, including third-trimester infection, admission to an intensive care unit, and the presence of secondary pneumonia accompanying the 2009 H1N1 infection [8]. Several studies have reported similar results [9]. The vaccination of pregnant women confers protection to the women against severe disease and to the newborns through transplacental antibodies during pregnancy [10], as well as after delivery via breastfeeding [11].

Despite the great efforts to increase the coverage of maternal immunization against influenza, this proves difficult [12]. Copious published data have described the effectiveness and safety of the vaccine [10,13,14,15,16]. Vaccine effectiveness against hospitalizations can range from 40 to 70% in different locations (40% in Australia, Canada, Israel, and the United States; 70% in Mali; and 50% in South Africa) [13,14,15,16]; nonetheless, vaccine coverage during pregnancy still has not reached the desired rates in the US and globally [17], and Mexico is not an exception, reporting a decrease in influenza vaccine coverage in this population from 81% (2018) to 65% (2020) [18,19]. Several factors contribute to this. A lack of proper knowledge regarding the disease and the vaccine as well as a lack of confidence in the effectiveness and safety profile may be some of the primary contributors to this problem [12]. 

In Mexico, influenza vaccination in pregnancy is a priority, and free access is available to all pregnant women that wish to acquire it. However, as a free vaccine for public settings, its availability is limited [20]. Nonetheless, robust national data regarding influenza vaccination rates by risk groups, influenza-related complications, and deaths during pregnancy are not well described or publicly available [21,22].

Mexican prenatal and obstetric care falls under the care of the Obstetricians (OBGYNs) and Family Physicians (FPs), who oversee the follow-up of the pregnancy. The national official Mexican norm (NOM-007-SSA) promotes that all pregnant women of low risk receive a minimum of five prenatal consultations and a maximum of eight consultations [23].

During prenatal care, the providers of obstetric care must take a complete immunization history, but many women do not recall or do not have a record of their lifetime vaccinations. Ideally, a woman should have her complete and updated vaccination schedule before pregnancy, and this would avoid any concern about completing it during pregnancy. 

We hypothesized that Mexican healthcare providers (HCP) for pregnant women (OBGYNs and FPs) are not aware of influenza complications during pregnancy (and for the newborn) or the benefits of influenza vaccination in this high-risk group.

The objective of this study was to assess the knowledge, beliefs, and practices of OBGYNs and FPs in Mexico regarding influenza disease and influenza vaccination during pregnancy, with the intent to define the current situation regarding disease awareness and educational gaps related to this topic.

## 2. Material and Methods

We conducted a descriptive study to evaluate the knowledge, beliefs, and practices of Mexican healthcare providers (OBGYNs and FPs) on influenza disease, seasonal influenza immunization of pregnant women, and their current recommendation through a survey. We focused on these HCPs as they are traditionally the ones in charge of maternity care in Mexico and as recommended by the Mexican Institute of Social Security (IMSS) [23]. 

We defined our study population using the following criteria: inclusion criteria: certified OBGYNs and FPs who provide prenatal outpatient care and are members of the Mexican Colleges of OBGYNs and FPs; exclusion criteria: uncertified OBGYNs and FPs that do not provide prenatal outpatient care and are not members of the Mexican Colleges of OBGYNs and FPs. Accordingly, all physicians belonging to the Mexican Associations of OBGYNs and FPs who complied with the inclusion criteria between 1 February and 31 March 2020 were included (when the influenza season practically ends). A sample size determination was therefore not performed, and the sample was obtained by convenience from all who agreed to participate. 

### Survey Design and Delivery

We used the Google Survey^®^ platform to create, send, and collect our questions and responses. The collection of responses occurred over two months (February–March 2020), and only the responses obtained during that period were considered for this study. 

The survey design was composed of 32 questions divided into four sections. Section one included the demographic characteristics of the population. All answers were multiple-choice. Section two included 11 questions on knowledge about seasonal influenza disease, clinical features, diagnosis, treatment, vaccination, and the impact on pregnant women. The answers were yes, no, or I do not know.

Sections three and four included a Likert-scale questionnaire [24] about beliefs and practices related to seasonal influenza vaccination in the pregnant population. We used both multiple-choice (yes, no, and unknown) and open questions.

We had support from the Mexican Associations of OBGYNs and FPs through their branches in the state of Baja California. They distributed our survey among their current members and colleagues (2098 members) in other states of the country on the condition of confidentiality.

We used Microsoft Excel^®^ to conduct a descriptive, qualitative, and mostly univariate analysis. We tabulated and analyzed all data by gender, medical specialty, years of experience, and place of work. An Odds Ratio (OR) analysis was conducted for each question to determine statistically significant differences among the responses of the OBGYNs and FPs. 

## 3. Results

The questionnaire was electronically sent to all 2098 members of the Mexican Associations of both OBGYNs and FPs, from which during the two-month period, we obtained a total of 206 complete surveys, representing a response rate of 9.8% and providing a total of 2472 daily consultations.

In Table 1, we describe the demographic characteristics retrieved from our study population. Similar distributions were obtained for the specialties (OBGYNs—47.6%, FPs—52.4%), most of the respondents were females (63.6%), and 65.4% of the responses came from the state of Baja California. The Mexican Institute of Social Security (IMSS), followed by private practice, were the primary medical practice institutions, at 37% and 34%, respectively.

As for their years of experience, the majority reported having between 1 and 5 years (56%). FPs reported a higher influenza vaccination rate than OBGYNs, at 83.3% and 66.3%, respectively.

All responses to the survey questions can be found in Table 2. Here, we highlight the most important findings. In total, 199 (96.6%) of our participants knew about seasonal influenza and its cause. Nevertheless, only 151 (73.3%) correctly identified the different types of influenza viruses that cause seasonal epidemics and outbreaks in humans; 20.4% responded that in their opinion the clinical picture of influenza disease is like a common cold, rarely severe, and does not cause death; 99% correctly identified the high-risk groups for influenza complications; and 10.1% stated that they were not aware that infection—mostly milder—could occur even if vaccinated.

Most participants (180/206; 86.4%) answered correctly that PCR is the standard tool to confirm influenza infection. With respect to treatment, 150 (72.8%) had the correct answer, and 56 (27.2%) did not. As for preventive measures, 197 (95.6%) of them correctly responded that vaccination is the best way to prevent infection and disease. 

In total, 152 (73.8%) respondents in our study’s population agreed that pregnant women are more frequently hospitalized due to influenza when compared with non-pregnant women, whilst 17% disagreed and 9.2% did not know. Moreover, 100 (48.5%) acknowledged that adverse effects due to influenza infection can affect the fetus, but 106 (51.5%) considered this to be false or did not know about it. In addition, 45 (22%) believed that contracting influenza disease and its complications are exclusive to pregnant women with concomitant comorbidities, such as diabetes, obesity, hypertension, and autoimmune diseases, among others.

As for the appropriate time to vaccinate a pregnant woman, 117 (56.8%) replied that vaccination can occur any time during pregnancy; 49 (23.8%) replied that it could take place during the second trimester, 27 (13.1%) replied that it could take place during the first trimester, and 13 (6.3%) replied that it could take place during the third trimester.

In the beliefs section, more than half (61%) responded that they either strongly disagreed, disagreed, or did not know that pregnancy alone is not a risk factor for developing influenza complications, but 39% reported the opposite. Only 22% agreed that influenza disease in a pregnant woman confers a health or developmental risk to the fetus, and a minority (6%) believed that “influenza vaccine is NOT safe and can cause influenza infection”.

More than half (66%) reported having treated a pregnant woman with influenza and believed that seasonal influenza disease can be harmful in this population. Conversely, the remaining 34% replied that they had not had contact with these cases, and their responses ignored the severity of the disease in this population. In total, 198 (96.1%) considered that immunization against influenza during pregnancy is a safe and effective preventive intervention. 

In total, 62 (30%) physicians believed that influenza vaccination only conferred protection to the mother, but not to the fetus, and this assumption was equivalent between OBGYNs (32.3%) and FPs (31.3%). In addition, 33 (16%) did not know whether influenza vaccination protected both the mother and the baby, a mistaken assumption that was more common in FPs (21.6%) than in OBGYNs (12.74%).

When responding to their actual clinical practices, 98.1% recommend influenza vaccination during pregnancy. In addition, 79.6% responded that they always (56.8%) or very often (22.8%) checked the vaccination status of their patients. As for the appropriate time during pregnancy when they feel comfortable recommending the influenza vaccination, 89.3% considered that this could be either in the first or second trimester (44.2% and 45.1%, respectively). The remaining 10.7% preferred the third trimester for vaccination.

**Figure 1:**
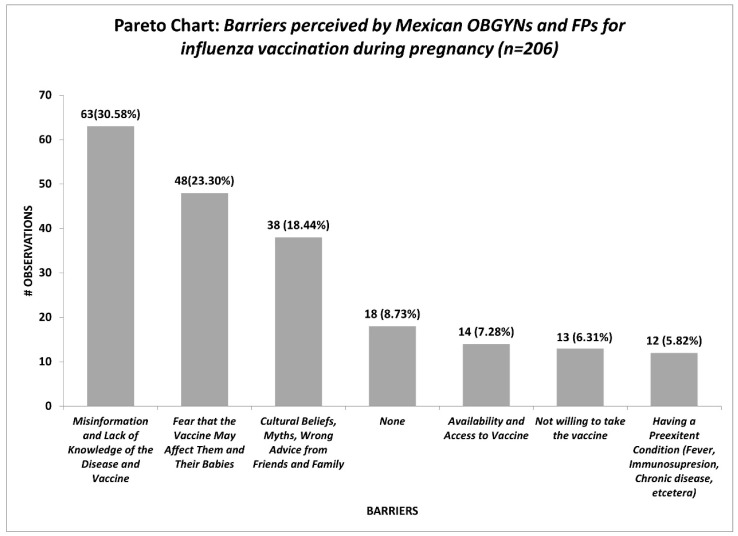
Reported perceived barriers by OBGYNs and FPs regarding influenza vaccination during pregnancy.

The reported barriers by OBGYNs and FPs concerning influenza vaccination during pregnancy are shown in Figure 1. Misinformation and a lack of knowledge of the disease and vaccine (30.58%); fear that the vaccine may affect them and their babies (23.3%); and cultural beliefs, myths, and wrong advice from friends and family (18.44%) represent the most significant barriers for the medical providers.

We performed an OR calculation for each question to determine whether statistically significant differences between OBGYNs and FPs were found. We did not find any. Moreover, we also performed the same statistical analysis in relation to provider vaccination status between OBGYNs and FPs, and by gender, and again, there were no statistically significant differences (see Table 3).

## 4. Discussion

To the best of our knowledge, this study is the first investigating the knowledge, beliefs, and practices associated with influenza vaccination during pregnancy in a group of maternity care providers in Mexico. OBGYNs and FPs are the cornerstones of healthcare for pregnant women in Mexico, and they play an essential role in the current recommendation and implementation of all preventive measures in pregnancy, especially vaccine uptake. 

Even though influenza vaccination in pregnant women has been implemented since 2004 in Mexico, it was not until 2009, after the AH1N1 influenza pandemic, that more significant efforts were made to actively recommend its use in this high-risk population [21,25]. Nevertheless, despite these efforts, influenza vaccine coverage among the pregnant population remains low in Mexico. Reports on national coverage suggest that influenza vaccine coverage is as low as 56.5% for the whole population; however, a recent publication indicated that vaccination coverage among pregnant women decreased from 81% in 2018 to 65% in 2020 [19,26]. 

In our study, the majority (98.1%) of the surveyed HCPs were aware and supportive of the current recommendations for influenza vaccination for pregnant women. The above discovery is consistent with the results of other studies from the US and Europe showing high rates of recommendation during pregnancy by physicians [27,28,29,30]. Nevertheless, in those same studies, they found that influenza vaccination among these medical providers was only 75%, despite their recommendation. 

The overall knowledge about influenza illness and vaccines was somehow high (79.46%) among both populations. Areas of opportunities for education are the impact of influenza infection on the fetus (51.5% incorrect); the safety of vaccination in pregnancy (42.8% incorrect); the timing of vaccination during pregnancy (43.2% incorrect); and that pregnancy is a risk factor for being hospitalized due to influenza complications (26.2% incorrect).

Based on these knowledge gaps, our responders might be recommending influenza vaccination with varying levels of clarity and conviction. Similar results have been documented in prior research, indicating that various prenatal care providers possess varying levels of knowledge regarding influenza vaccination during pregnancy [27,28,29,30]. The latter is noteworthy, as increased levels of knowledge are linked with higher rates of influenza vaccination in pregnant women [30].

We also noted that the reported knowledge was being contradicted in some areas in the beliefs section. For example, whilst 99% of the HCPs knew the high-risk groups, in the beliefs section, 48% reported not considering pregnancy as a high-risk factor and 22% believed that complications due to influenza among pregnant women only occur in those who have chronic conditions, such as obesity, diabetes mellitus, hypertension, and cardiac disorders, among others.

Although the increased risk of severe complications and mortality of influenza infection during pregnancy is well documented for both the expectant mother and the fetus [16], this remains a critical gap in the knowledge of our responders since 22% replied that no risk was conferred to the fetus when the mother develops influenza. We assume this gap in knowledge might be linked to the fact that 34% acknowledged never treating a pregnant woman with influenza and therefore doubt the severity of this disease in this population. Esposito et al. [31] reported similar outcomes, where 40.6% of the OBGYNs acknowledged not considering influenza as a potentially severe disease.

It is imperative that prenatal care providers are aware of the implications of influenza infection in utero, as it causes five times more perinatal mortality. Furthermore, recent publications have indicated that exposure to influenza in utero is linked to slight declines in mid-childhood mental health, primarily driven by an elevation in the internalizing symptoms of mental health. Additionally, for males, there are increased depressive symptoms observed in mid-life as a result of this in utero exposure to influenza [32].

Another discrepancy that was noted was the vaccination timing during pregnancy: 58.6% reported in the knowledge section that any time is appropriate to do so, but 75.8% answered the same question correctly in the beliefs section. The biggest difference came from answers related to knowledge vs. beliefs regarding the protection induced by vaccination: 30% believed that the vaccine only confers protection to the mother but not to the baby. However, 96.1% responded in the beliefs section that the vaccine is an effective measure to prevent influenza, reduces the risk of hospitalization (92.8%), and is safe and effective (94%) for both the mother and the newborn. These results reflect a clear disconnect between what physicians know and believe when it comes to influenza vaccination during pregnancy, highlighting that knowledge on its own does not always drive clinical practices.

The variability in the responses makes us infer that our study population does not possess a clear understanding of the safety profile of the influenza vaccine or the impact of the severity of influenza disease on the pregnant population. They possess good theoretical knowledge; however, when implementing that knowledge into their daily practices, their practices are not in line with what they know. Efforts are needed to align their knowledge with their beliefs and practices, as this denotes a barrier in proper recommendations. The main reported barriers from the HCP perspective regarding the uptake of the influenza vaccine in their pregnant populations were misinformation and a lack of knowledge of both the disease and the vaccine (63/206; 30.58%) and the fear expressed by pregnant patients to their HCPs that the vaccine may affect them and their babies (48/206; 23.3%). In addition, sociocultural beliefs, myths, and wrong advice from friends and family have a strong influence on patients accepting immunization.

Initially, we were expecting that the main barrier was that the pregnant woman did not want the vaccine; however, this potential barrier to vaccination was reported by 13/206 (6.31%) of our respondents. 

Similar findings were reported by Maisa et al. [33], where information and knowledge were the primary barrier, followed by the influence of others, acceptance and trust in both the HCPs and the vaccine, fear and distrust, responsibility for the baby, and lastly access to vaccination.

As a final point to this, perceived barriers are essential to be identified since, as HCPs or even as patients, we often think that access to care and willingness are the main barriers when they are the opposite; they just represent the tip of the iceberg. Conducting an appropriate root cause analysis of this problem using different tools for continuous process improvement is something that should be compulsorily promoted.

Our study has limitations: The geographic distribution of the responders is not representative of the HCP population distribution in Mexico; it is a small sample; and selection bias cannot be excluded, as there was no control on whom the societies contacted. Our response rate was low (9.8%), as only 206/2098 HCPs responded during the 2-month period. Future studies need to consider expanding the scope to include more HCPs from different states and medical institutions. The distribution of medical specialties and gender was not representative of the current situation in Mexico either.

However, even with a relatively small sample of physicians, the estimated 2472 daily pregnancy consultations among our responders give significant strength to our study. We report valuable information on where to direct the efforts to improve vaccine uptake in pregnant women since our HCPs have a vast area of opportunity to reach a large number of patients.

## 5. Conclusions

Our results highlight an evident discrepancy in knowledge vs. beliefs regarding influenza disease and vaccination during pregnancy for physicians caring for pregnant women.

These findings underline that knowledge alone is not sufficient to make proper recommendations for influenza vaccination during pregnancy. Hence, understanding the drivers of beliefs and attitudes will be essential to ensure a more effective method to educate HCPs, resulting in improved clinical practice and recommendations from OBGYNs and FPs to all pregnant women, potentially leading to enhanced influenza vaccine uptake in pregnancy. 

Merely presenting factual information does not guarantee the sustained adoption of long-term health behavior changes. Hence, it is imperative not only to continuously monitor and evaluate all programs aimed at educating healthcare professionals about influenza vaccination but also to implement better and wider educational programs for influenza and influenza vaccination in pregnancy directed to both OBGYNs and FPs as part of their yearly certification to foster enduring transformations in their practices.

## Figures and Tables

**Table 1:** Demographic characteristics.

Demographics of Study Population (n = 206)		
Medical Specialty	OBGYNs	98 (47.6%)
	FPs	108 (52.4%)
Gender	Female	131 (63.6%)
	Male	75 (36.4%)
Geography/State	Baja California	134 (65.4%)
	Mexico City	20 (9.3%)
	Other states	52 (25.3%)
Primary Medical Practice Institution	Mexican Institute of Social Security (IMSS)	76 (37%)
	Private Sector	69 (34%)
	Secretary of Health (SSa) Sector	31 (15%)
	2 or more institutions	30 (14.5%)
Years of work experience	1–5 years	115 (56%)
	6–10 years	29 (14%)
	>10 years	62 (30%)
Completed influenza immunization	OBGYNs (n = 98)	65 (66.3%)
	FPs (n = 108)	90 (83.3%)
Average number of daily pregnancy consultations	12	Translated into 2472 daily pregnancy consultations among our study population.

**Table 2:** Knowledge, beliefs, and practices of HCPs regarding influenza vaccination during pregnancy in Mexico.

Questions	Responses	All	(%)
Knowledge Section			
Seasonal influenza disease is an acute respiratory infection caused by the influenza virus, which circulates throughout the world?	True False I do not know	199 5 2	(96.6) (2.4) (1)
There are 4 types of seasonal influenza viruses, A, B, C, and D. But only type A and B viruses are responsible for seasonal outbreaks and epidemics of the disease.	True False I do not know	151 31 24	(73.3) (15) (11.7)
The clinical picture of Influenza disease is like the common flu, is rarely severe, and does not cause any death.	True False I do not know	164 42 0	(79.6 (20.4) (0)
All age groups can be affected by seasonal influenza. However, there are higher risk groups that develop the disease and its complications. Such are: Children under 5 years of age, pregnant women, older adults, individuals with chronic diseases.	True False I do not know	204 1 1	(99) (0.5) (0.5)
The onset of seasonal influenza symptoms may occur suddenly or progress more slowly, and patients who received the vaccine may have milder symptoms.	True False I do not know	185 13 8	(89.8) (6.3) (3.9)
The standard criterion for confirming influenza virus infection remains a polymerase chain reaction with reverse transcription (PCR) or viral culture of nasopharyngeal or throat secretions.	True False I do not know	178 15 13	(86.4) (7.3) (6.3)
Antiviral medications for seasonal influenza include oseltamivir, zanamivir, and baloxavir marboxil.	True False I do not know	150 37 19	(72.8) (18) (9.2)
The most effective way to prevent infection and disease is through annual vaccination against the Influenza virus of all individuals at risk.	True False I do not know	197 9 0	(95.6) (4.4) (0)
Pregnant women tend to be frequently hospitalized due to the development of more severe illness and development of complications, and they can even die due to seasonal influenza, than women who are not pregnant.	True False I do not know	152 35 19	(73.8) (17) (9.2)
Some of the adverse effects of influenza infection in the fetus are the restriction of fetal growth, fetal distress, neural tube defects, preterm birth, increased fetal mortality.	True False I do not know	100 55 51	(48.5) (26.7) (24.8)
When is the best time to vaccinate pregnant women against seasonal influenza?	Any Trimester 1st 2nd 3rd	117 49 27 13	(56.8) (23.8) (13.1) (6.3)
Beliefs Section			
Pregnancy per se is NOT a risk factor for developing influenza disease and its complications.	Strongly Agree Agree Neutral Disagree Strongly disagree	47 34 18 43 64	(22.8) (16.5) (8.7) (20.9) (31.1)
Influenza disease does NOT confer any health or developmental risk to the fetus.	Strongly Agree Agree Neutral Disagree Strongly disagree	14 17 15 82 78	(6.8) (8.3) (7.3) (39.8) (37.9)
The influenza vaccine is NOT safe and can cause influenza infection.	Strongly Agree Agree Neutral Disagree Strongly disagree	1 1 11 63 130	(0.5) (0.5) (5.3) (30.6) (63.1)
I have never treated a pregnant patient with influenza, so doubt the severity of this disease in this population.	Strongly Agree Agree Neutral Disagree Strongly disagree	16 27 27 55 81	(7.8) (13.1) (13.1) (26.7) (39.3)
Do you consider that vaccination against the influenza virus in pregnant women is a SAFE and EFFECTIVE practice to prevent the disease?	Strongly Agree Agree Neutral Disagree Strongly disagree	155 43 5 2 1	(75.2) (20.9) (2.4) (1.0) (0.5)
Only pregnant women with chronic degenerative diseases (DM, HBP, Lupus, etcetera.) are prone to contracting Influenza disease and its complications.	Strongly Agree Agree Neutral Disagree Strongly disagree	22 19 4 66 95	(10.7) (9.2) (1.9) (32) (46.1)
Influenza vaccination in pregnant women only gives protection to the mother, but not to the baby.	Strongly Agree Agree Neutral Disagree Strongly disagree	22 19 4 66 95	(10.7) (9.2) (1.9) (32) (46.1)
Pregnant women who were vaccinated against influenza reduce their risk of being hospitalized due to complications.	Strongly Agree Agree Neutral Disagree Strongly disagree	133 58 4 7 4	(64.6) (28.2) (1.9) (3.4) (1.9)
The Influenza vaccine can be applied in pregnant women during any trimester of pregnancy.	Strongly Agree Agree Neutral Disagree Strongly disagree	113 43 13 25 12	(54.9) (20.9) (6.3) (12.1) (5.8)
Practices Section			
Do you recommend and promote influenza vaccination during pregnancy?	Yes No	202 4	(98.1) (1.9)
Do you offer influenza vaccination among all your patients regardless of their pregnancy status (pregnant or not pregnant)?	Yes No	177 29	(85.9) (14.1)
Usually during which pregnancy trimester do you promote or apply influenza vaccination?	1st 2nd 3rd	91 93 22	(44.2) (45.1) (10.7)
Do you check the immunization status of all your patients?	Always Very often Sometimes Rarely Never	117 47 26 5 11	(56.8) (22.8) (12.6) (2.4) (5.3)
Would you be interested in learning more about the benefits of influenza vaccination during pregnancy?	Yes No	198 8	(96.1) (3.9)
What challenges or barriers do you consider play a role in influenza vaccine uptake during pregnancy?	Open question		

**Table 3:** Influenza vaccination status among providers and OR analysis (n = 206).

Odds Ratio Analysis of Provider Vaccination Status						
	OBGYNs	FPs			Female	Male
YES	65	90		YES	100	55
NO	33	18		NO	31	20
	(OR = 1.25, 95% CI = (0.82–1.91), *p*-value = 0.28)				(OR = 0.96, 95% CI (0.62–1.48), *p*-value = 0.85)	

## Data Availability

Since this was an anonymous survey, all data was saved in an excel file as mentioned in the methodology. Data is unavailable due to privacy restrictions.
